# Enhanced word learning and neural synchrony during children’s storytelling

**DOI:** 10.1038/s41539-026-00417-7

**Published:** 2026-03-28

**Authors:** Nina Besser Ilan, Hadas Shavit, Nofar Zohar, Sagi Jaffe-Dax

**Affiliations:** 1 Sagol School of Neuroscience, Tel Aviv University, Tel Aviv, Israel; 2 Faculty of Psychology, University of Vienna, Vienna, Austria; 3 School of Psychological Sciences, Tel Aviv University, Tel Aviv, Israel; 4 The Miriam and Aaron Gutwirth Medical School, The Weizmann Institute of Science, Rehovot, Israel; 5 Baruch Ivcher School of Psychology, Reichman University, Herzliya, Israel

**Keywords:** Neuroscience, Psychology, Psychology

## Abstract

Children learn better through shared social experiences like storytelling, where they benefit as both listeners and tellers. Encouraging children to tell stories is a form of scaffolding, an instructional strategy in which learners and instructors are actively engaged. While the educational benefits of storytelling and scaffolding are well-documented, their underlying neural processes remain underexplored. Shared experiences are generally reflected in neural synchrony, which is often linked to comprehension. This study examined learning outcomes and neural synchrony in young school-aged children engaged in storytelling through either scaffolding or passive listening. Results showed that scaffolding improved learning outcomes and was associated with higher synchrony between teacher and young learner. However, these learning benefits disappeared in remote learning environments, highlighting the importance of face-to-face interaction for active learning strategies. Taken together, the heightened cortical synchrony between teacher and learner in face-to-face settings points to the benefits of active interaction as a learning strategy.

## Introduction

For young children, learning novel words and their functions depends highly on social engagement with adults and other children^1,2^. This engagement often occurs through storytelling, where children can be both listeners and tellers. While storytelling is considered an effective learning strategy, little is known about the neural mechanisms underlying this shared experience in children. The current study addressed this gap by investigating the neural synchrony between an adult and a child during a storytelling session. Specifically, we compared two types of learning strategies in the context of storytelling: passive learning, in which the story is told to the child, and scaffolding-based learning, where the child is encouraged to tell the story.

Throughout history, stories have been used to impose order on human experience^3^. Especially during childhood, stories and storytelling provide a framework for thinking and sharing knowledge, and create a meaningful connection between children and their environment^4^. By leveraging the social nature of language^2^, storytelling supports language acquisition and enriches children’s oral language complexity, grammar, vocabulary, and sentence formation, while also improving reading comprehension and writing proficiency^5–7^. Furthermore, storytelling helps children understand complex concepts such as those required in science and mathematics. For instance, Hu et al.^8^ found that preschool-aged children could grasp basic astronomy concepts through personification storytelling. Storytelling also supports narrative skills, emergent literacy, and social competence^9^, improves listening comprehension in second language learners^10^, enhances problem-solving skills^11^, and promotes creativity^12^.

While children benefit from listening to stories, they also gain a great deal by being storytellers themselves. Whether recounting personal experiences or retelling familiar stories, the act of storytelling enhances children’s narrative skills and contributes to developing a stronger sense of self^13–15^. Research suggests that encouraging children to take on the role of storyteller during shared book reading^16^, create their own stories^17^, and share their personal narratives^13,15^ can be seen as part of a scaffolding process. Scaffolding is an effective instructional strategy^18–22^ in which instructors use constructive engagement behaviors, such as asking key questions, modeling, and providing hints and feedback^23^, to help learners generate ideas that extend beyond the provided material by relating it to prior experience. This turn-taking dynamic, in which both participants are active^24,25^, enriches learning with additional meanings, rationales, and justifications, thus potentially fostering deeper understanding^26^. The concept of scaffolding is rooted in Vygotsky’s sociocultural theory, which posits that learning initially occurs on a social level before it becomes internalized^27^. Specifically, scaffolding derives from Vygotsky’s concept of the Zone of Proximal Development (ZPD); i.e., the distance between what children can do independently and what they can do with the support of a more knowledgeable other^27–29^.

In the context of storytelling, when adults encourage children to tell stories, ask questions about them and give feedback, they provide verbal support and a narrative structure that can enhance children’s narrative skills and improve their memory of the experience^13,16,17^. The scaffolding effect may be even more pronounced when children are encouraged to tell a story related to a learning topic. Since story creation is a constructive process, it allows children to build new knowledge from their existing understanding and imagination. Moreover, collaborative story creation enables teachers to tailor the learning experience to individual learners’ needs while modeling the learning subject in a way that is both engaging and familiar. However, it is unclear whether storytelling is more effective when children actively construct a story about a learning topic compared to passively listening to a story and whether these different social learning experiences are supported by distinct neural mechanisms.

Several neuroimaging studies have begun to characterize the neural activity associated with social experiences, including storytelling and scaffolding, by measuring brain activity in relation to the activities of other participants. This approach, known as “second-person neuroscience”, argues that since social interactions give rise to many cognitive faculties, a complete understanding of cognitive processes requires examining brain activity during the entire course of interactions in relation to others’ brain responses^30–32^. Research has shown that shared experiences are reflected in neural synchrony between participants (inter-subject correlation^33^). Such synchrony has not only been found in low-level perception regions but also in social cognition and higher-order regions, including the default mode network (DMN^34,35^), attention networks^36^ and extralinguistic networks^37,38^. Synchrony in these regions is thought to reflect shared understanding, as it diminishes when participants listen to nonsensical or temporally scrambled speech, or to narratives in an unfamiliar language^37–39^. Moreover, several of these regions overlap with the mentalizing network, which is engaged during the inference of others’ mental states and is believed to support understanding of others’ goals and intentions^34,40–42^.

Researchers implement two main approaches to study the mechanisms underlying neural synchrony during shared experiences. The first is designed to examine the neural synchrony between multiple individuals perceiving the same social stimulus (e.g., listening to a shared story), and aims to quantify the extent of similarity in the representations of external social inputs across different brains^33,39,43–45^. The second examines synchrony between two or more individuals during direct interactions or concurrent experiences, in what is known as brain-to-brain coupling^30,31,38,39,46,47^. Most research on social dynamics between individuals adopts the latter approach, as it allows for the examination of the dynamics between both sides of the interaction. The responses in the listener’s brain often lag behind those of the speaker, suggesting that responses in the speaker’s brain causally shape the responses in the listener’s brain^37,47,48^. This finding, however, is not consistent across studies^46,49^. Additionally, some studies have reported that the extent of coupling is linked to mutual gaze^50,51^. Notably, studies have found that neural coupling is linked to successful learning^46,52–56^.

Despite the vast neuroimaging research on neural synchrony during shared learning experiences^51,55,57,58^, the neural mechanisms of storytelling-based learning in children remain underexplored. Recent studies have begun to address this topic. For instance, in their pivotal fNIRS storytelling study, Piazza et al.^33^ found that preschoolers could acquire a range of semantic information from listening to a story, and showed that child-child neural synchrony in the parietal cortex predicted word learning. Similarly, in a shared book reading experiment, Zhai et al.^59^ demonstrated that mother-child brain coupling in the superior temporal cortex was associated with positive effects on preschoolers’ language ability. Interestingly, the directionality of time-lagged neural synchrony between the two participants reversed during the interaction, potentially reflecting bidirectional information flow. Zhang et al.^60^ reported that teaching science concepts through storytelling was correlated to higher child-teacher neural synchrony in the left and right inferior frontal gyri compared to traditional teaching methods, and that synchrony in the left supramarginal gyrus was correlated to learning outcomes. Despite these initial efforts, research on brain-to-brain coupling in childhood has largely focused on mother-child synchrony during cooperation tasks^61–63^ or on adult-infant dynamics^50,64^, rather than on storytelling-based learning. The situation is similar in adult research. Although numerous studies have examined speaker-listener neural coupling, they often rely on prerecorded narratives^37,39^ or focus on conversations and lectures^49,52^, rather than on naturalistic storytelling.

The neural mechanisms underlying scaffolding have also been recently addressed. In their fNIRS study, Pan et al.^55^ compared a scaffolding-based strategy with an explanation-based strategy that focuses on clarifying information to enhance comprehension^65^. Their findings indicated that scaffolding not only led to better learning outcomes than the explanation-based approach, but was also linked to increased instructor-learner brain-to-brain coupling in prefrontal regions. The authors suggested that this enhanced coupling reflected the intersubjectivity and communicative turn-taking dynamic intrinsic to the scaffolding process. However, this study focused on adults, thus limiting its applicability to children’s scaffolding experiences and the corresponding neural mechanisms involved. Moreover, it remains unclear whether these mechanisms extend to storytelling-based learning and whether they similarly relate to learning outcomes.

To address this gap, we investigated the learning benefits and neural mechanisms of scaffolding within the context of storytelling. Specifically, we compared children’s performance when they actively created a story (scaffolding storytelling) with their performance when they passively listened to one and examined whether these strategies are associated with different patterns of neural coupling during social interaction. Based on Pan et al.^55^, we hypothesized that a scaffolding-storytelling strategy would result in better learning outcomes and higher brain-to-brain coupling than a passive story-listening strategy.

**Fig. 1:**
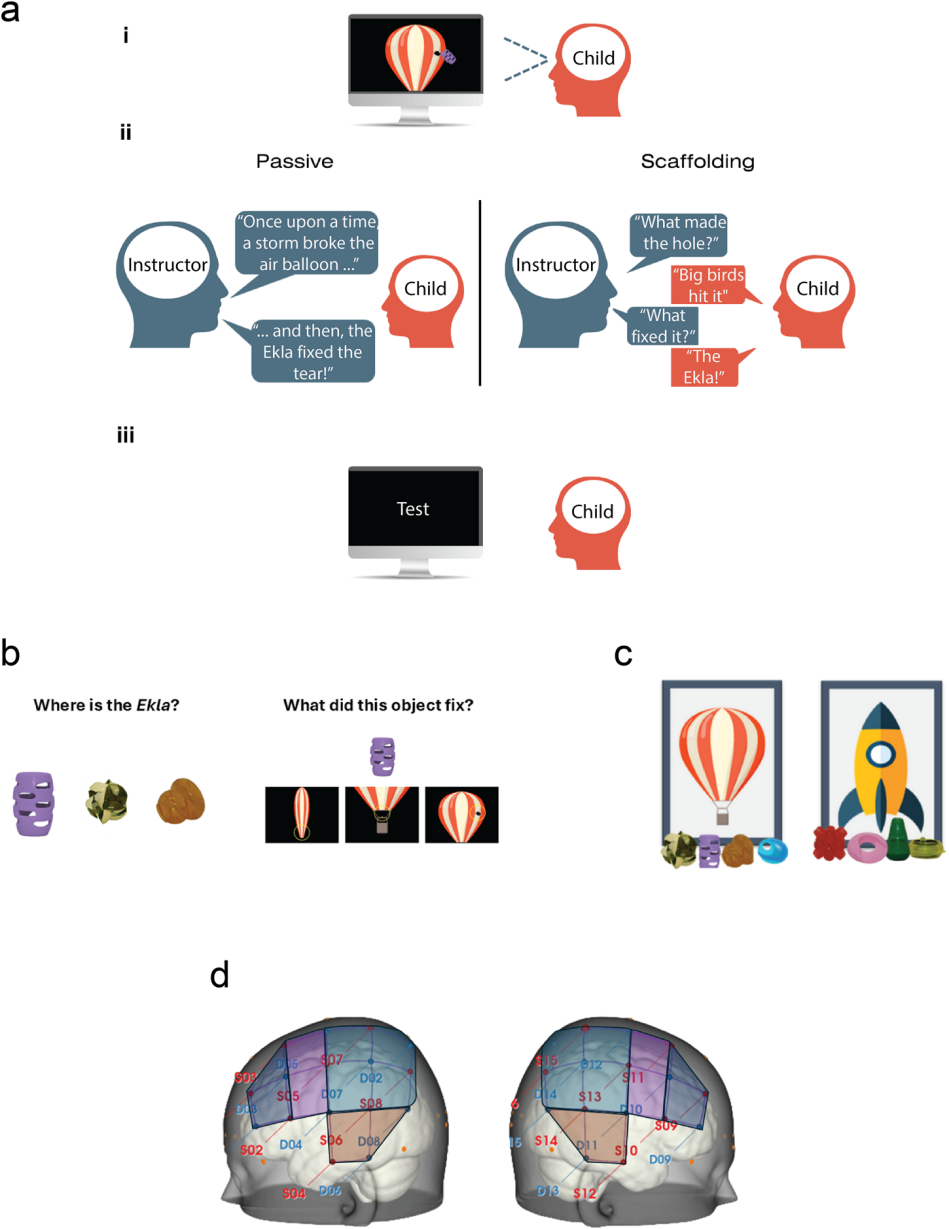
Experimental protocol and probe locations. **a** Experimental procedure: Children watched a video presenting stimuli (**i**), participated in an interactive session with an instructor (**ii**), and were tested on the learned materials (**iii**). Stimuli and strategy (Scaffolding or Passive) were randomly assigned. The procedure was repeated with the other condition and stimuli set. **b** Question types. The questions on the test were about the objects’ names (“Where is the [object’s name]?”) and functions (“What did this object fix?”). **c** Sets of stimuli. **d** Regions of interest. Eight in total, left and right: prefrontal cortex (*dark blue*), premotor cortex (*purple*), temporal lobe (*brown*), and parietal lobe (*light blue*).

First-grade-age children took part in two interactive learning sessions with an instructor who engaged in passive story-listening or scaffolding storytelling (Fig. 1). Each interactive learning phase was preceded by a brief video of the learned materials, which were unfamiliar novel objects, and was followed by a test on the names of the novel objects and their functions. Using fNIRS, we recorded neural activity in the frontal lobes, the temporal lobes, and the parietal lobes of the child and the instructor. We conducted a child-instructor synchronization analysis (within-session) and a child-child synchronization analysis (across-sessions).

Six hypotheses were formulated:

1. Scaffolding storytelling would lead to better learning than passive storytelling.
2. Learning should be driven by the interactive phases, such that the correlation between the activity of children who viewed the same video (child-child neural synchrony) would be overall positive but would not predict learning outcomes.
3. Consistent with Pan et al.^55^, the Scaffolding strategy would be associated with higher child-instructor neural synchrony than the Passive strategy.
4. In both strategies, child-instructor synchrony would engage regions that are associated with social learning (e.g., the prefrontal cortex, the temporoparietal junction, superior/inferior parietal lobe, the premotor cortex^34,35,66–68^). However, we expected the brain regions and the overall synchrony patterns to differ as a function of learning strategy^55,57,58^. These patterns may reveal correlations between synchrony and mutual gaze^50,51^. Given mixed evidence in the literature, no specific regions were hypothesized for each strategy: prior studies have associated storytelling with either prefrontal^60^ or superior temporal coupling^59^, and active versus passive learning with either prefrontal differences^57^ or more widespread frontotemporal-parietal engagement^55^.
5. In line with previous studies on speaker-listener dynamics, in the Passive condition, the brain activity of the instructor (speaker) would consistently precede the brain activity of the child (listener). Directionality in the Scaffolding condition was treated as exploratory, given the absence of fixed speaker–listener roles in the participants’ mutual exchange.
6. In line with previous studies, child–instructor neural synchrony during the interactive sessions would be directly correlated to children’s learning outcomes.

In addition to the face-to-face experiment, we conducted a supplementary remote version of the task to examine whether the behavioral effects of scaffolding storytelling depend on in-person interaction. This question is particularly relevant given the widespread adoption of remote learning environments following the COVID-19 pandemic. Although remote learning offers important advantages, including flexibility in time and location^69^, most research has reported reduced learning outcomes in online settings^70,71^, often due to difficulties in sustaining learner engagment^72,73^. In contrast, other studies suggest that remote learning can yield comparable performance when teaching is instructor-led^74^ and when sufficient interaction is maintained^75^. Given the potential of scaffolding storytelling to support learner engagement, it is important to examine whether its possible effect extends to online contexts.

## Results

### Storytelling is an effective learning strategy that benefits from scaffolding

**Fig. 2:**
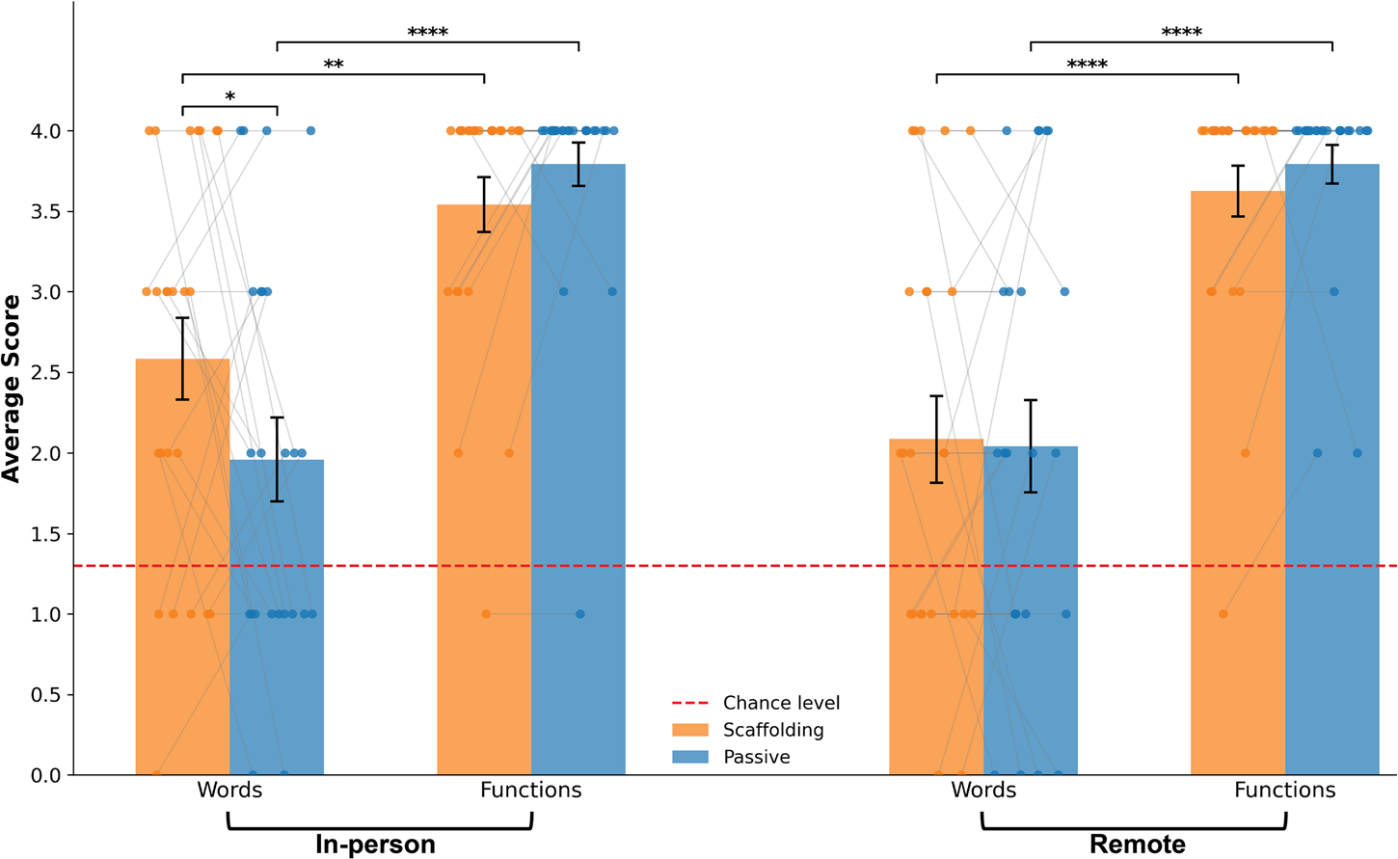
Performance by learning strategy and question type in in-person and remote environments. Each test included 8 questions: 4 on object names (Words) and 4 on object functions (Functions). The maximum score was 4 for each question type (max test score = 8). The overall learning was above chance for both strategies (3 alternative answers on each question), with higher performance for object functions than for names [In-person – Scaffolding: *t*(23) = 3.4, *p* < 0.005; Passive: *t*(23) = 6.2, *p* < 0.001; Remote – Scaffolding: *t*(23) = 5.6, *p* < 0.001; Passive: *t*(23) = 5.9, *p* < 0.001]. Scaffolding showed a significant advantage over Passive in word learning [*t*(23) = 1.8, *p* < 0.05]. This advantage was not observed in the remote experiment. *N* = 24.

For both conditions, overall learning was significantly above chance [Scaffolding: 6.1 ± 1.6, *t*(23) = 10.43, *p* < 0.001, Cohen’s *d* = 2.13; Passive: 5.7 ± 1.4, *t*(23) = 10.61, *p* < 0.001, Cohen’s *d* = 2.16, one sample *t*-test]. Learners’ performances were better for object functions than for object names in both conditions [Scaffolding: 3.5 ± 0.8 vs. 2.6 ± 1.2, *t*(23) = 3.4, *p* < 0.005, Cohen’s *d* = 0.70; Passive: 3.8 ± 0.6 vs. 1.9 ± 1.2, *t*(23) = 6.2, *p* < 0.001, Cohen’s *d* = 1.3]. Contrary to our hypothesis, learning strategy did not affect the learners’ total scores [6.1 ± 1.6 vs. 5.7 ± 1.4, *t*(23) = 0.9, *p* = 0.2], probably due to a ceiling effect in learning the object functions (Fig. 2). Nevertheless, the learning strategy had a significant effect on question type scores [*F*(1, 23) = 5.8, *p* < 0.05, partial *η*^2^ = 0.20, repeated measures ANOVA], with Scaffolding showing a significant advantage in word learning (object names) compared to the Passive strategy [2.6 ± 1.2 vs. 1.9 ± 1.2, *t*(23) = 1.8, *p* < 0.05, Cohen’s *d* = 0.4; Fig. 2]. The Scaffolding strategy thus boosted performance on the more difficult task.

### Better learning in Scaffolding is not driven by greater stimulus exposure or child speech production

Notably, words were better learned in the Scaffolding condition despite the instructor repeating the object names more often in the Passive condition [11.5 ± 3.5 vs. 19.1 ± 2.4 times, *t*(22) = −6.5, *p* < 0.001, Cohen’s *d* = 1.3]. Furthermore, scaffolding elicited better word learning even though children spent significantly more time looking at the cards illustrating the stimuli in the Passive condition [68.8 ± 9.5% vs. 38.5 ± 11.6%, *t*(22) = −11.62, *p* < 0.001, Cohen’s *d* = −2.42], and looked at the stimulus together with the instructor more often in the Passive condition than in Scaffolding [20.6 ± 4.9% vs. 13.7 ± 6.2%, *t*(22) = −5.06, *p* < 0.001, Cohen’s *d* = −1.05]. Importantly, subjects’ overall scores were not predicted by neither the overall frequency with which the instructor mentioned the object names and functions [Scaffolding: *r*(22) = −0.07, *p* = 0.7; Passive: *r*(22) = −0.002, *p* = 0.99], nor the length of the learning session [which was comparable across conditions: 151.8 ± 19.9 s vs. 150.4 ± 9.6 s, *t*(23) = 0.4, *p* = 0.7; Scaffolding: *r*(23) = -0.17, *p* = 0.4; Passive: *r*(23) = 0.34, *p* = 0.1]. Similarly, there were no significant correlations between learning outcomes and stimulus-gaze measures [child gaze on stimulus – Scaffolding: *r*(22) = 0.38, *p* = 0.23, Passive: *r*(22) = 0.29, *p* = 0.28; joint gaze on stimulus – Scaffolding: *r*(22) = 0.27, *p* = 0.28, Passive: *r*(22) = 0.25, *p* = 0.28]. The gaze analysis also detected no boredom behaviors in either condition. All results were FDR-corrected^76^.

To further understand the mechanisms underlying the scaffolding advantage, we performed turn-taking analyses in the Scaffolding condition. On average, the instructor produced 15.4 ± 3.4 speaking segments, while children produced 13.5 ± 3.2. Mean segment duration was 5.0 ± 1.4 s for the instructor and 4.3 ± 1.5 s for children. Children accounted for 38.9 ± 9.2% of the total talk time, corresponding to an average speaking duration of 55.7 ± 14.4 s. The instructor asked an average of 1.04 ± 0.17 questions per segment, which children answered at a rate of 0.90 ± 0.15 per segment. Child-initiated questions were rare (child-to-instructor: 0.01 ± 0.03 per segment). Despite the verbal participation, learning outcomes did not correlate with measures of child speech production, including average segment length [*r*(22) = 0.13, *p* = 0.83], speaking ratio [*r*(22) = 0.20, *p* = 0.83], or total speaking time [*r*(22) = 0.04, *p* = 0.85]. Moreover, the advantage of Scaffolding in word learning occurred despite children rarely producing the object names themselves during these sessions (0.61 ± 1.1 times), instead predominantly mentioning the objects’ functions (3.6 ± 2.1 times; *t*(22) = −5.9, *p* < 0.001, Cohen’s *d* = −1.24). All results were FDR-corrected^76^.

### Child-child ISC during video watching is positive and unrelated to learning

**Fig. 3:**
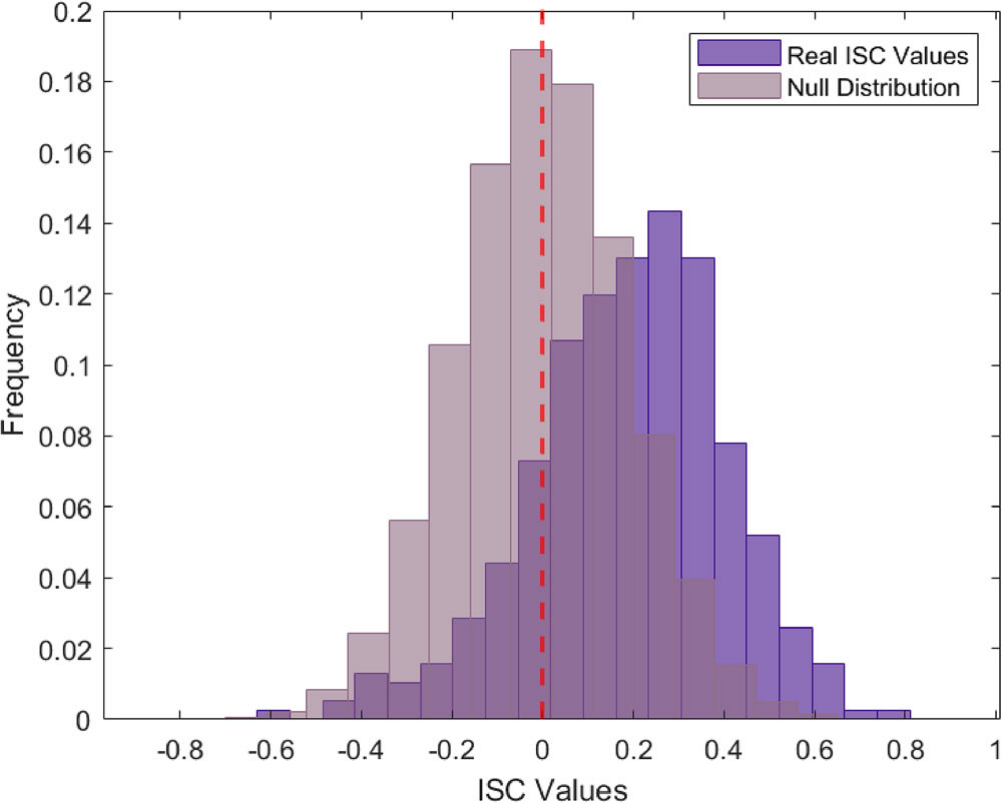
Histogram of child-child inter-subject correlation (ISC) during video watching. Pearson correlations between the brain activity of each subject and the averaged brain activity of the group [excluding them] was calculated for each ROI in each video. The histogram presents the Fisher's *Z* values of these Pearson values from both videos (ISC scores) as well as the null distribution of the phase-scrambled signals. *N* = 24.

In both videos, the children’s brain activity in each region of interest (ROI) was positively correlated with the group-averaged brain activity (Fig. 3; all results were FDR corrected^76^). This finding is consistent with our hypothesis and supports other studies that have reported inter-subject correlations (ISC) in higher-order regions between subjects who watched the same stimuli^44,77^. As expected, our results did not indicate a significant correlation between children’s performance and their ISC values in any ROI (Fig. S2).

### Child-instructor neural synchrony is overall higher in scaffolding storytelling than in passive story-listening

**Fig. 4:**
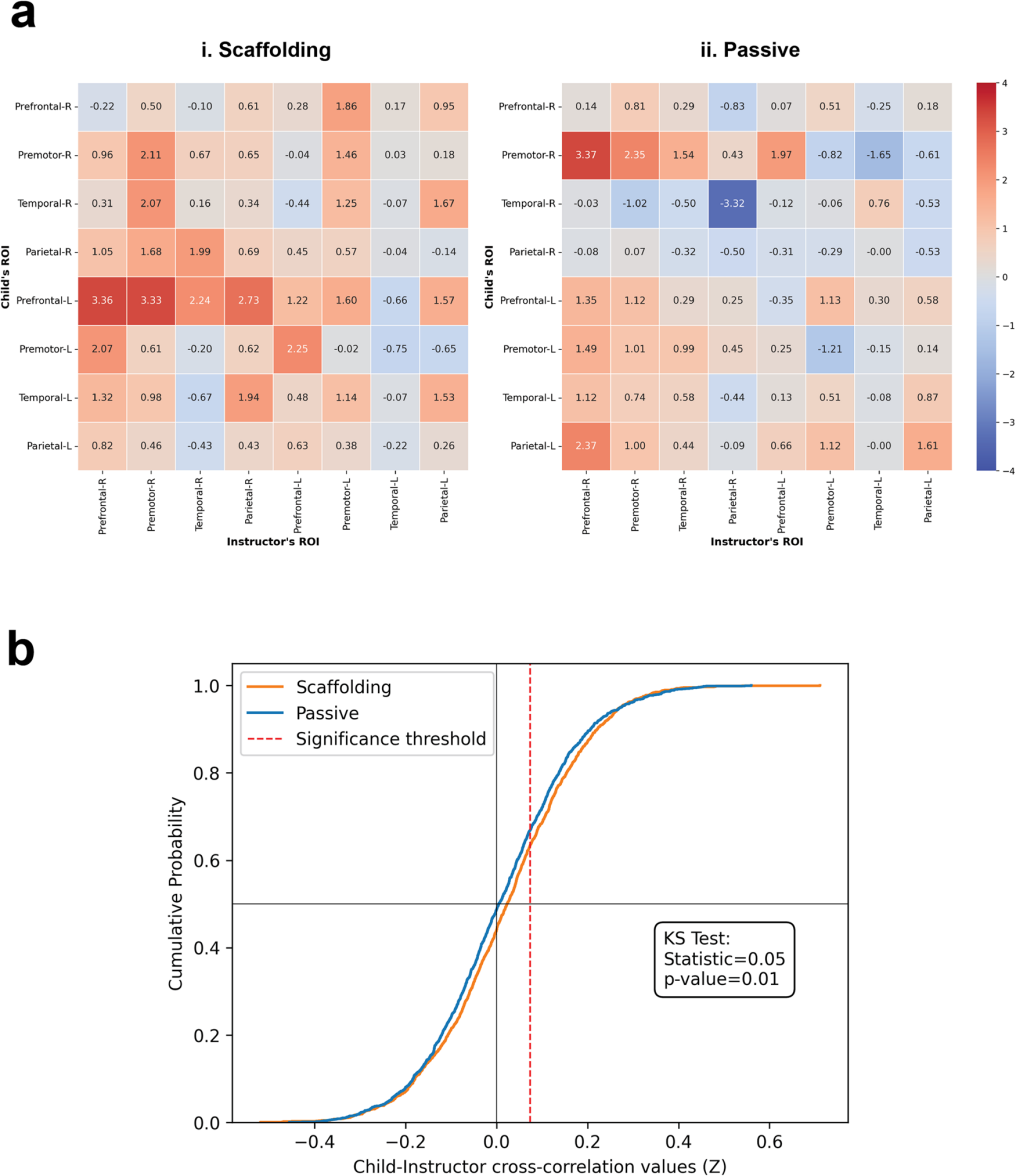
Child-instructor synchrony by learning strategy. **a** Child-instructor neural synchrony during Scaffolding **(i)** and Passive **(ii)** sessions. Rows and columns represent children’s and instructor’s ROIs, respectively (R - right, L - left). The values shown are *t*-statistics from one sample *t*-tests comparing the Fisher’s *Z* values to zero. **b** Cumulative distribution functions (CDFs) of the Fisher’s *Z* values in Scaffolding (orange) and Passive (blue) sessions. The correlations between the child’s brain activity and the instructor’s brain activity were calculated for each ROI pair for each dyad (1536 pairs of ROIs). The x-axis shows the Fisher’s *Z* values, and the y-axis shows the probability of obtaining a correlation value or lower. The dashed red line represents the threshold value for a significant positive correlation (*p* < 0.05) with degrees of freedom ranging from 700 to 800 – the average range for the degrees of freedom in the child-instructor data. The black lines denote the center of a null distribution (cumulative probability of 0.5 and synchrony of 0). Overall, neural synchrony during the scaffolding sessions was higher than during the passive sessions. The distribution of synchrony values was different for the two learning strategies (*KS* = 0.05, *p* = 0.01, Kolmogorov-Smirnoff test for equal distributions).

Overall, the child-instructor neural synchrony during the Scaffolding sessions was higher than in the Passive sessions (Fig. 4a). A comparison of the cumulative distribution functions (CDFs) of the Fisher’s *Z* values indicated that Passive sessions had higher cumulative probabilities for lower correlation values than the Scaffolding sessions. A Kolmogorov-Smirnov test for equal distributions (Fig. 4b) confirmed that the correlation values were significantly higher in the Scaffolding sessions than in the Passive sessions (*KS* = 0.05, *p* = 0.01).

### Child-instructor synchrony has a distinct pattern for each learning strategy

**Fig. 5:**
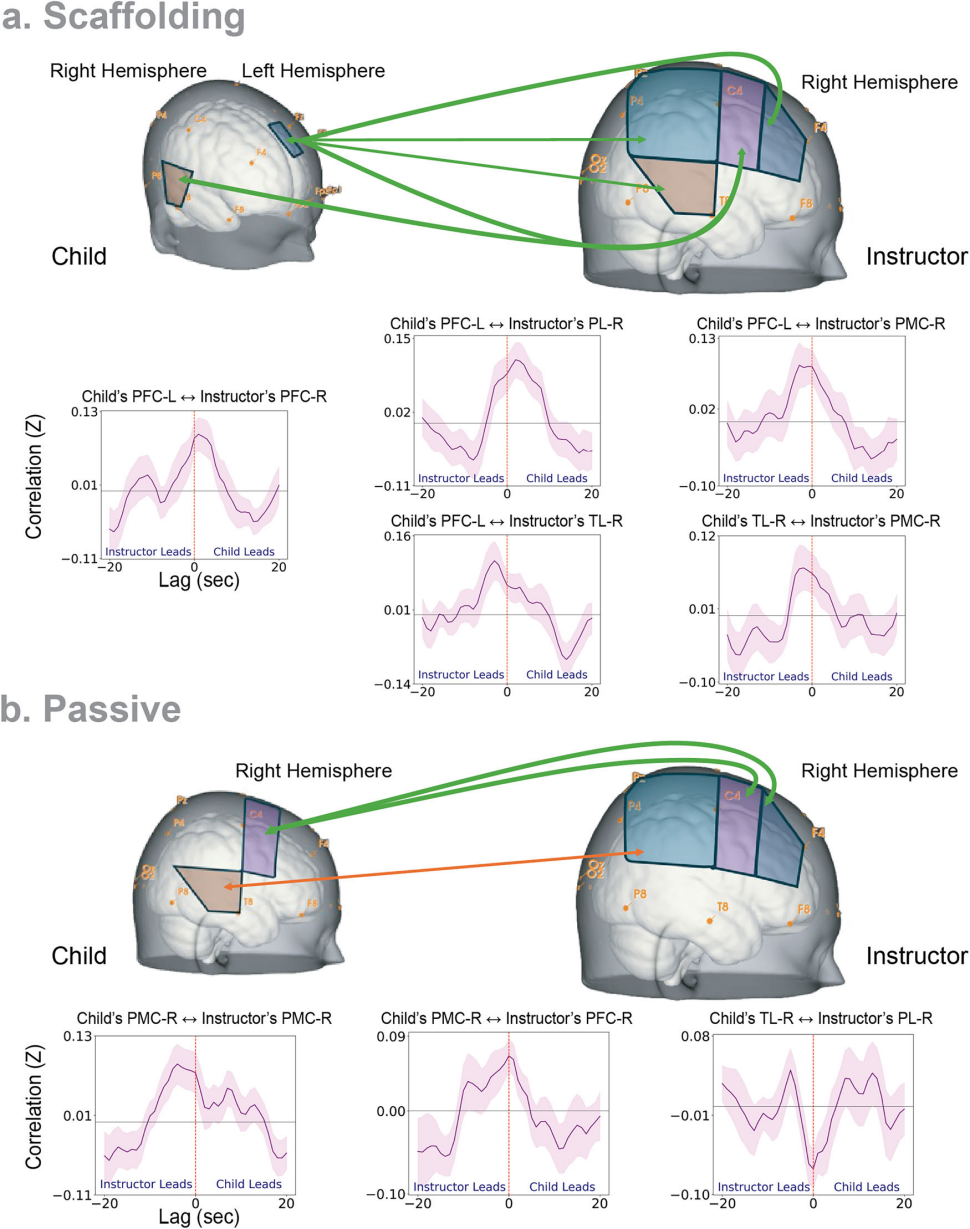
Child-instructor neural synchrony patterns by learning strategy. The correlation between each child’s and instructor’s ROIs [left and right: prefrontal cortex (PFC) – dark blue, premotor cortex (PMC) – purple, temporal lobe (TL) – brown, and parietal lobe (PL) – light blue; *N* = 24] in Scaffolding (**a**) and Passive (**b**) sessions. Significant correlations (*p* < 0.05, permutation testing, *n* = 1000) are indicated by arcs, green for positive and orange for negative correlations. The average child-instructor correlation across all dyads is shown in lag plots. The x-axis shows the time shift (in seconds) between the child and the instructor. Positive values indicate that the child is ahead of the instructor, and negative values indicate that the instructor is ahead of the child. The dotted red line indicates neutral time (lag = 0). In the Scaffolding sessions, the instructor led in most coupled ROIs (with peaks occurring just before lag = 0), whereas in the Passive sessions, this only occurred in one ROI pair.

During the Scaffolding sessions, the neural activity of the children and the instructor was coupled in five pairs of ROIs (Table 1, Fig. 5a). Interestingly, this synchrony was mainly between the children’s left prefrontal cortex and the instructor’s right hemisphere ROIs. For the Passive sessions, however, the child-instructor neural synchrony only occurred in three pairs of ROIs, all of which were in the right hemisphere of the child and the instructor (Table 1, Fig. 5b). This synchrony mainly involved the children’s right premotor cortex. There was negative synchrony between the child’s activity in the right temporal lobe and the instructor’s activity in the right parietal lobe. These patterns of synchrony differed overall between the two learning strategies. For the majority of the dyads, the Euclidean distance between their correlation map and the group-averaged map was smaller when the group-averaged map was from the same condition and was classified correctly (*d’* = 1.28; *p* < 0.05).

**Table 1:** Significant child-instructor neural synchrony by ROI and lagged correlation

	Child’s ROI	Instructor’s ROI	*t*(23)	Lag (sec)	Leader
Scaffolding	Prefrontal-L	Prefrontal-R	3.4*	+1	Child
	Prefrontal-L	Premotor-R	3.3*	−3	Instructor
	Prefrontal-L	Temporal-R	2.2*	−3	Instructor
	Prefrontal-L	Parietal-R	2.7*	+2	Child
	Temporal-R	Premotor-R	2.1*	−2	Instructor
Passive	Premotor-R	Premotor-R	2.3*	−4	Instructor
	Premotor-R	Prefrontal-R	3.4***	0	None
	Temporal-R	Parietal-R	−3.3***	0	None

*R* right, *L* left

*p < 0.05, ***p < 0.001, permutation testing, *n* = 1000

This analysis was also conducted for the HbR concentrations, where Scaffolding coupling was observed in six ROI pairs. However, no significant coupling was found in the Passive sessions (Fig. S1, results are shown without correction for multiple comparisons). This aligns with studies indicating that HbO more reliably reflects brain-to-brain coupling^37,55^.

### Instructor leads neural responses in most regions in Scaffolding but only one in Passive

In line with our hypothesis and previous studies, the neural response of the child (listener) lagged behind the instructor (the speaker) in the Passive condition. This result might be interpreted as indicating that the instructor causally shaped responses in the child’s brain while leading the interaction. However, this pattern was only observed when the premotor cortex of the child and the instructor were coupled, with the correlation peaking at a negative lag of 4 s. There was no lag correlation in the other two coupled regions (Table 1, Fig. 5b). In contrast, the neural response of the children lagged behind the instructors in most regions in the Scaffolding condition, with the correlation peaking at a negative lag of 2–3 s (Table 1, Fig. 5a). However, when the child’s left prefrontal cortex was coupled with the instructor’s right parietal and right prefrontal cortex, the child’s neural responses preceded those of the instructor (the correlation peaked at a positive lag of 1–2 s).

### No direct correlation between neural synchrony and children’s scores

Contrary to other studies, we did not find a direct correlation between child-instructor neural synchrony and learning outcomes in any of the coupled ROIs that showed significant correlations (Fig. S3). This finding held true for both strategies and remained consistent when focusing specifically on word learning scores. Given the finding that child-instructor synchrony lagged in time, we examined whether adjusting for this lag in synchrony, optimized for each significantly coupled ROI, could predict learning outcomes. However, even with this adjustment, neural synchrony was still not directly correlated to learning outcomes (Fig. S4).

### Mutual gaze predicts prefrontal cortex synchrony in Scaffolding sessions

**Fig. 6:**
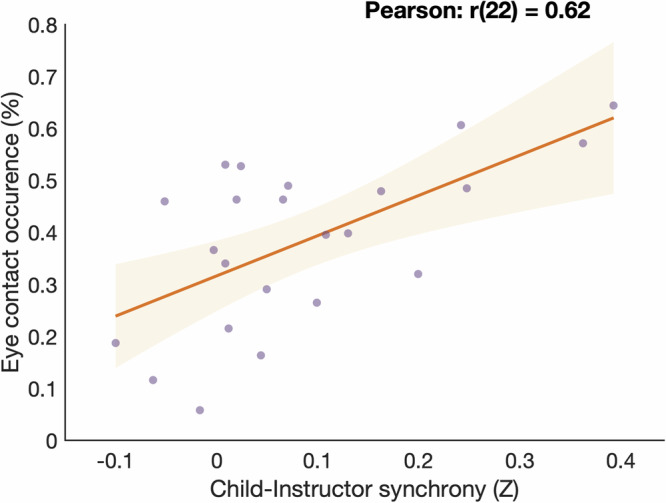
Percentage of mutual gaze predicts the correlation between the child’s left prefrontal cortex and the instructor’s right prefrontal cortex in Scaffolding sessions. Cortical synchrony between these ROIs was predicted by mutual gaze (*r* = 0.62, *p* < 0.05). *N* = 23.

During both interactive sessions, the instructor and the child had mutual eye contact during more than one-third of the interactions, with no significant difference between strategies [Scaffolding: 38.4 ± 16.0%, Passive: 40.5 ± 16.3%, *t*(22) = −0.73, *p* = 0.46]. For both strategies, there was no correlation between the percentage of eye contact and the children’s performance [Scaffolding: *r*(22) = −0.37, *p* = 0.23, Passive: *r*(22) = -0.15, *p* = 0.5]. However, interestingly, for the Scaffolding strategy alone, there was a correlation between child-instructor neural synchrony in the significantly coupled ROIs and the percentage of mutual gaze. Specifically, there was a strong correlation between mutual gaze and coupled activity of the child’s left prefrontal cortex and the instructor’s right prefrontal cortex [*r*(22) = 0.62, *p* < 0.05, all results were FDR corrected^76^; Fig. 6]. No significant associations were observed between neural synchrony and stimulus-directed gaze or turn-taking measures in any of the significantly coupled ROIs.

### Scaffolding advantage disappears in remote learning

In the remote experiment, the overall learning was similar to the learning in in-person setting [Scaffolding: 5.7 ± 1.6 vs. 6.1 ± 1.6, *t*(23) = -0.97, *p* = 0.3; Passive: 5.8 ± 1.6 vs. 5.7 ± 1.4, *t*(23) = 0.3, *p* = 0.8; Fig. 2] and was significantly above chance [Scaffolding: *t*(23) = 8.99, *p* < 0.001, Cohen’s *d* = 1.83; Passive: *t*(23) = 9.67, *p* < 0.001, Cohen’s *d* = 1.97, one sample *t*-test]. Similar to the in-person setting, children’s performance was better for object functions than for object names in both conditions [Scaffolding: 3.6 ± 0.7 vs. 2.1 ± 1.3, *t*(23) = 5.6, *p* < 0.001, Cohen’s *d* = 1.14; Passive: 3.8 ± 0.6 vs. 2.0 ± 1.4, *t*(23) = 5.9, *p* < 0.001, Cohen’s *d* = 1.2], and learning strategy did not affect the children’s total scores [*t*(23) = −0.71, *p* = 0.7].

Interestingly, the better recall in Scaffolding vs. Passive word learning was not found in the remote setting. The learning strategy had no significant effect on question type scores [*F*(1,23) = 0.3, *p* = 0.59, repeated measures ANOVA], with the Scaffolding condition yielding similar performances for object names as the Passive condition [2.1 ± 1.3 vs. 2.0 ± 1.4, *t*(23) = 0.13, *p* = 0.89]. This occurred despite the fact that Scaffolding sessions were slightly longer than Passive sessions [Scaffolding: 143.2 ± 13.7 s vs. Passive: 135.8 ± 6.9 s, *t*(23) = 3.3, *p* < 0.005, Cohen’s *d* = 0.67]. This finding suggests that a physical in-person experience is crucial for the learning benefits of Scaffolding to emerge.

## Discussion

This study explored the neural mechanisms and learning outcomes associated with child-instructor storytelling sessions. The findings indicated that while children can learn from both Passive and Scaffolding storytelling strategies, the Scaffolding approach was more effective in terms of the acquisition of novel words during physical in-person interactions *(h1)*. Importantly, the better learning in scaffolding was not driven by greater exposure to the learned stimuli and was not correlated to child’s speech production. The shared storytelling experiences were reflected in the neural synchrony between the child’s and the instructor’s higher-order and social cognition brain regions, with Scaffolding eliciting greater and more extensive neural coupling *(h3)*. However, neural synchrony was not directly correlated to individual learning outcomes *(h6)*, and neither was mutual eye contact. The results further revealed that Passive and Scaffolding storytelling differed in their patterns of neural synchrony *(h4, h5)*. In Scaffolding storytelling, child-instructor synchrony primarily involved the child’s left prefrontal cortex, lagged in time with the instructor typically leading, and was predicted by mutual gaze. In contrast, in Passive story-listening, synchrony predominantly engaged the child’s right premotor cortex, mostly did not lag in time, and was not predicted by mutual gaze. These patterns were distinct, implying that the neural mechanisms associated with storytelling differed when the child was encouraged to tell a story as compared to simply listening to one. These differences may reflect fundamental distinctions in the educational structure and approaches of the two strategies.

To the best of our knowledge, this is the first study to examine brain-to-brain coupling in children learning novel words within a social learning context. While some studies have explored how child-parent coupling dynamics during shared book reading relate to language ability^59^, and others have examined child-instructor coupling during science learning sessions that were structured around storytelling^60^, the current study specifically focuses on the dynamic child-instructor neural interactions during a structured word learning experience. This again constitutes the first direct comparison of child-instructor neural synchrony during scaffolding and passive storytelling in middle childhood learning. By analyzing brain-to-brain coupling across both conditions, we provide insights into the ways in which different interaction dynamics influence the neural mechanisms of learning novel words in children. The findings extend current understanding of the role of storytelling in learning by showing that scaffolding storytelling not only enhances active engagement but also reshapes the temporal dynamics of neural synchrony, which may reflect the more responsive and interactive learning environment.

Consistent with previous studies, we found that better learning outcomes were associated with higher levels of neural synchrony in social cognition brain regions^48,55^. The superiority of Scaffolding as a learning strategy aligns with Interactive-Constructive-Active-Passive (ICAP) theory^26^, which posits that constructive engagements, such as creating a story based on learned information under the instructor’s guidance, are more effective than passive engagements (e.g., listening to the instructor’s story). In this constructive process, both participants actively develop a shared understanding through communicative exchanges, allowing children to learn from the instructor and deepen their understanding^23^. Importantly, these bidirectional exchanges were correlated to learner-instructor degree of coupling observed in adult research^55^. Therefore, greater neural synchrony in Scaffolding may reflect the cognitive and communicative processes engaged during constructive interactions, which support children’s comprehension and learning.

Mutual engagement during Scaffolding is further supported by behavioral analysis of turn-taking dynamics. While the instructor generally led the sessions – producing more talk segments, asking at least one question per segment on average, and speaking more overall – the child was actively engaged, contributing a substantial number of talk segments, responding to nearly every question, and speaking for more than a third of the session. Interestingly, the results of the lagged synchrony analysis may reflect this turn-taking pattern. Each participant’s brain activity led in some coupled regions of interest (ROIs) while following in others, suggesting dynamic communication and role-switching^25,59^. Nevertheless, in most regions, the instructor’s brain activity preceded the child’s activity, which might be interpreted as an indication that the instructor primarily shaped the responses of the child’s brain while leading the interaction^37,47,48^. Although this may seem counterintuitive, given that the child actively generated the story, it aligns with key features of the Scaffolding strategy; namely, the contingent support principle^29^ and the learner’s reflective role and constructive behaviors^25,26^. To tailor the learning experience to the child, the instructor must stay a step ahead, while the child reflects on new information and integrates it with prior knowledge^23^.

The advantage of Scaffolding in word learning may be reflected in the extensive involvement of the child’s left prefrontal cortex in neural synchrony during these sessions. This region includes Broca’s area, which is associated with phonological and phonetic processing^78^. Moreover, the prefrontal cortex supports cognitive control, attention, and goal-directed behaviors^79^, suggesting that the bidirectional exchange of information in scaffolding may engage it more strongly. The involvement of the child’s prefrontal cortex is consistent with recent studies that have identified similar coupling exclusively during scaffolding^55^ as well as research highlighting the role of the prefrontal cortex in social interactions^34,35,66–68^, including brain-to-brain fNIRS studies examining social interactions in children and infants^50,61,80^. Specifically, the observed neural synchrony between the child’s left prefrontal cortex and the instructor’s right prefrontal cortex is aligned with research on neural dynamics in participants engaging together to achieve a shared goal^61,81,82^. Neural coupling between these regions was predicted by the percentage of mutual gaze, which is consistent with infant studies where it was shown to be associated with joint attention^50,83^. Taken together, these findings may point to the relation between neural synchrony and the active engagement of both instructor and learner, as they collaboratively build a shared understanding.

Unlike the active participation that characterizes Scaffolding, Passive story-listening places the child in a primarily receptive role. This fundamental difference might be reflected in the unique neural synchrony patterns of the Passive condition – most notably, the involvement of the child’s right premotor cortex. This involvement aligns with studies reporting the role of the premotor cortex in inter-subject correlations during social learning^51^, as well as storytelling^38^ and story-listening experiences^40,45^. Those studies have suggested that the premotor cortex contributes to higher-level linguistic processes^45^, including action-perception circuits at the syntactic and semantic levels^38^, and to differentiating intentions and beliefs behind nonmotor behaviors^40^. Moreover, the premotor cortex is known for its part in perceiving and predicting others’ actions within goal-directed contexts, which is essential for social learning^66,67^. The broader engagement of the premotor cortex during Passive listening compared to Scaffolding may therefore reflect its role in forming internal representations of the narrative as the speaker describes actions and intentions. Shared neural representations have been linked to shared narrative understanding in both speaker-listener and listener-listener contexts^77^, such that individuals who understand a character’s actions similarly exhibit more similar neural responses than those who understand the narrative differently^40,84^. This interpretation of premotor engagement is supported by the finding that the instructor’s neural activity preceded the child’s when their right premotor cortices were coupled. The instructor’s leadership is consistent with research on speaker-listener coupling during storytelling^37,47,52^, and reflects a “sender-receiver” dynamic^30,34^, where the instructor’s actions are perceived by the child, creating interdependent brain activity patterns^85,86^. Thus, the instructor’s leading neural activity may help the child anticipate the plot and foster greater mutual understanding^40,66,87^.

However, no lagged correlations were found in the other two coupled regions during the Passive sessions, suggesting that the instructor’s influence on the child’s neural responses was overall weaker in the Passive condition than in Scaffolding. One potential explanation is that the instructor was less engaged, possibly due to boredom from repeating a scripted story. Nevertheless, this argument is undermined as a similar pattern was observed in a study where the speaker was recorded once, and no lag was found between the speaker’s and listeners’ neural responses while listening to the recording^46^. A more plausible explanation is that the children were less engaged in the Passive sessions, which could be reflected in their lower scores for word learning in comparison to scaffolding sessions. This assumption aligns with ICAP theory, which argues that passive learning is less engaging and less effective than constructive learning^26^. Notably, this reduced engagement was not observed in the behavioral coding: no boredom-related behaviors were spotted during Passive sessions, and measures of attention, such as eye contact and gaze toward the stimulus^51^, were comparable to or even higher than in the Scaffolding condition. Based on these attentional measures alone, learning would be expected to be similar or superior in the Passive condition. Together, these findings strengthen the argument that differences in learning effectiveness were not driven by attentiveness to the stimuli per se, but rather by the presence of constructive engagement behaviors, which were absent in the Passive condition.

The role of child engagement becomes particularly important in light of the finding that the scaffolding advantage disappeared in the remote experiment. One possible explanation is reduced child engagement under these settings. This view is consistent with evidence showing that remote learning is generally less effective than in-person learning for children^88^, although it contrasts with studies demonstrating comparable outcomes in remote, instructor-led settings when sufficient interaction is maintained^74,75^. Due to technical constraints, neural and behavioral measures were not collected in the remote condition, limiting direct assessment of engagement. Future work could therefore test whether reduced engagement during remote scaffolding is reflected by decreased child-instructor neural synchrony and whether reduced eye contact contributes to this effect.

The findings for Passive storytelling also revealed novel negative coupling involving the child’s right temporal lobe. While neural synchrony in this region was present in both sessions, consistent with studies highlighting temporal areas in inter-subject correlations during social interactions (e.g., the temporoparietal junction^49,89^, the superior temporal cortex^46,48^, and Wernicke’s areas^45,47^), in the Passive sessions this region showed a negative correlation with the instructor’s right parietal lobe. This mirrors findings by Simony et al.^90^, who reported negative inter-subject correlations during story-listening between these regions. Similar to their study, our results indicated fluctuating synchrony with both negative and positive correlations over time. This suggests that Passive storytelling may involve complex neural coupling that could reflect a dynamic balance in attentional resources or cognitive engagement between participants. Further research is needed to understand the mechanisms behind these negative correlations and their possible impact on learning outcomes.

While the comparison between Scaffolding storytelling and Passive story-listening showed that greater neural coupling was associated with better learning outcomes, no direct correlation was observed within each condition. This is notable given that correlations between neural synchrony and learning have been observed across multiple neuroimaging methods, including fMRI^46,53^, EEG^52^ and fNIRS^48,54,60^. Several methodological and behavioral factors may explain why a direct relationship was not observed in the present study. First, regarding data analysis, averaging signals from multiple channels into one ROI may have lost critical data. Further, correlating the averaged data from the entire session with learning outcomes may have limited our ability to capture important temporal fluctuations in synchrony over time. Notably, fNIRS studies that have successfully predicted learning outcomes focused on synchrony at specific time points between individual channels^48,54,55^. However, we chose to average signals to ensure reliable data collection in children, who experience signal disruption. Second, regarding behavioral measures, our findings are limited to novel word learning due to a ceiling effect in learning object functions, likely due to the use of simple, familiar actions (e.g., opening a door). The reduced variability in behavioral scores may have limited statistical power, decreasing the likelihood of detecting a significant correlation. Future research could address this by using made-up actions (e.g., an object is “zarning” a door) or more complex functions, such as transforming the composition of the door (e.g., solid to gas).

At the same time, the absence of a direct correlation between neural synchrony and learning invites alternative interpretations. Although Scaffolding was associated with both increased learning and greater child-instructor coupling, the present data do not allow us to disentangle whether neural synchrony directly drove learning outcomes, or whether both effects emerged independently from other features of the Scaffolding interaction (e.g., the child’s more active engagement, the instructor’s contingent responsiveness to the child). A potential counter-argument is that the higher neural synchrony observed in the Scaffolding condition reflects the fact that the instructor was listening to the child. This interpretation aligns with prior work suggesting that brain-to-brain coupling may be linked to brain-to-speech locking and speech predictability^91–93^. However, speech predictability was inherently higher in the Passive condition, where the instructor followed a structured script, whereas Scaffolding involved spontaneous dialogue. If brain-to-brain coupling was primarily a function of predictability, higher synchrony would be observed in the Passive condition; however, this is inconsistent with our findings. Nevertheless, since adults are generally better at predicting than children^94,95^, it remains possible that the higher neural synchrony in the Scaffolding condition reflects the instructor’s active prediction of the child’s speech, which may be more robust than the child’s prediction of the instructor. This interpretation could also account for the finding that the instructor’s neural activity preceded the child’s in most regions. While we cannot entirely rule out this possibility, our results remain consistent with active-versus-passive learning designs in adults. In those studies, where participants have comparable predictive abilities, greater neural synchrony is consistently found during active learning and correlates with improved outcomes^55,96^.

Another limitation of this study is that the experimental conditions varied along multiple dimensions: both the storyteller (adult vs. child) and the level of interaction differed, with scaffolding occurring only when the child told the story. A more complete design would have two additional conditions: one in which the child tells a scripted story, and one in which the child scaffolds the instructor to tell a story. Such a design would also help disentangle the role of speech predictability from that of instructional strategy, as it would allow comparison of child-instructor neural synchrony across two “instructor-as-listener” situations, clarifying whether synchrony differences reflect listening alone or the active versus passive nature of the interaction. However, this approach is not feasible with children of this age, as they cannot reliably tell long, scripted stories or follow structured scaffolding instructions. A further limitation is the use of a single instructor, which may limit generalizability and introduce instructor-specific effects. Nevertheless, this approach aligns with prior instructor-learner and developmental studies^43,50,52,97,98^. Importantly, no performance differences were observed between early and late sessions, and the instructor reported no awareness of the study hypotheses, providing evidence against potential engagement declines or biases arising from adjustments in teaching behavior.

Future studies could extend this work by examining our findings in diverse learning environments. As previously noted, it would be beneficial to examine our design in remote learning contexts while analyzing gaze behaviors and child-instructor synchrony. Furthermore, since scaffolding can be effectively employed by educators^24,99–101^, it would be valuable to replicate our design in classroom settings with small groups, as has been done in studies with adults^52,97,98^ and adolescents^43^. This could also allow investigation of whether practices such as encouraging each group member to create a story around a novel word influence peer understanding and group neural synchrony^49^. Another noteworthy direction would be to explore the involvement of the premotor cortex in shared learning experiences, given its role in storytelling-related coupling.

This study contributes to the growing but still limited literature on brain-to-brain coupling between adults and children in middle childhood. Research on adult-child synchrony has primarily focused on infancy and early childhood, leaving middle childhood relatively underexplored compared to the extensive work on adult-adult synchrony^102^. This knowledge gap is particularly pronounced in learner-instructor dynamics^58^. However, as noted by recent frameworks, more examination is required to fully address the developmental aspects of child-adult neural dynamics. In the absence of longitudinal research, there is insufficient knowledge regarding how neural synchrony mechanisms change over time and experience and how they link to developmental variables and long-term outcomes^64,103^.

Together, these future directions could help map how interactive learning experiences influence neural coupling across childhood and contribute to a deeper understanding of how distinct social interactions shape middle childhood learning and its underlying neural mechanisms.

## Methods

### Participants

A sample size of 24 participants was selected based on previous studies examining learner-instructor neural synchrony in social interactions, which used equivalent (dyads *n* = 24)^48,55,104^ or similar (dyads *n* = 27)^51,57^ sample sizes. Consistent with prior teacher-learner research^46,53,97^, a single instructor was used. Thirty-three children were recruited for this study, all of whom were accompanied by their parents. Assent was obtained from the children before their participation, and their parents signed an informed consent form. The study and procedure were approved by the Ethics Committee of Tel Aviv University (IRB: 3606). The experiment was conducted in the testing room of the Cognitive Development Lab at Tel Aviv University. Parents were allowed to sit behind their child in the experimental room out of their line of sight. Nine subjects were excluded from the final data analysis: five for technical problems, three because they were in second rather than first grade, and one due to suspicion of cognitive disability. The remaining 24 participants (age 6.9 ± 0.3 years, 14 females) were included in the analysis. The instructor was an undergraduate psychology student who remained blind to the experiment’s purpose and hypotheses. Participants were native Hebrew speakers of Jewish-Israeli descent and came from middle-to-upper socioeconomic backgrounds.

### Procedure

Children learned two sets of stimuli –one in each strategy. Each set was composed of four novel objects that were presented as being tools to repair a rocket ship (adapted from Piazza et al.^33^) or an air balloon (Fig. 1c). In each condition, the children first watched a two-minute video showing how the tools were used to repair the rocket or the air balloon, and then engaged in an interactive session with the instructor (Fig. 1a). During the interactive session, the instructor employed either a passive strategy (telling the child a scripted story about the stimuli) or a scaffolding strategy (encouraging the child to create their own story about it). To enhance the storytelling experience, the instructor presented the child with a picture of the vehicle from the video pasted on oaktag, four cards illustrating the four stimuli, and a finger puppet of a dragon or an owl, which was introduced as the owner of the ship or the balloon. For each subject, the instructor’s strategy, the set of stimuli, and the finger puppet were selected randomly. In the Scaffolding condition, the instructor introduced the task by explaining that the owner’s vehicle was broken and prompted the child to create a story by asking guiding questions about how the parts might have been broken and how they could be repaired (Fig. 1a). Beyond this initial prompt, scaffolding was unscripted and adapted to the child’s narrative. While a few children required no additional support, most received further guidance from the instructor through specific behaviors: (1) open-ended questioning (e.g., “What could make the hole in the air balloon?”); (2) hinting (e.g., “We need to fix the hole in the air balloon; remember which of these tools does it?”); and (3) validation/feedback (e.g., “Exactly, the [object name] fixed the tear!”). In contrast, in the Passive condition, the instructor followed a fixed script and the child’s engagement was limited to silently listening to the story. After each interactive session, the children completed a three-alternative forced-choice questionnaire to measure their novel word learning. The test had eight questions, four on the names of the tools, and four on how the tools were used (their functions; Fig. 1b). The order of conditions was counterbalanced across children and the learning sessions did not differ in length [1^st^ session: 149.0 ± 13.9 s vs. 2^nd^ session: 153.2 ± 16.8 s, *t*(23) = -1.1, *p* = 0.3]. During the experiment, the children’s and the instructor’s brain activity was recorded simultaneously by fNIRS-based hyperscanning.

The videos were presented on a 43” screen positioned about 1.6 m from the child using PsychoPy. The quizzes were created by FlexiQuiz (https://www.flexiquiz.com). The sessions were recorded with a Logitech Brio 4k camera that was positioned above the screen. Due to a technical issue, one subject’s sessions were not captured on camera. The recordings of the 23 remaining subjects were annotated offline to identify behavioral gaze, turn-taking dynamics and the number of times the instructor and the child mentioned the object names and functions.

### Materials and stimuli

The stimulus objects, which the children were unlikely to be familiar with, were selected from the Novel Object and Unusual Name (NOUN) database^105^. The stimuli in each set had similar familiarity scores (21.3 ± 12.4% vs. 21.5 ± 13.3%) and were comparable to each other within the same set as well as to the stimuli in the other set (the similarity was based on the Euclidean distance, 0.55 ± 0.11 vs. 0.55 ± 0.13; 0.48 ± 0.12). Some objects had their colors altered using Adobe Photoshop software to reduce color similarity. The randomly assigned names for the objects were Hebrew pseudowords (e.g., “Ekla”^106^). The rocket ship illustration was adapted from Piazza et al.^33^, and the air balloon illustration was sourced from Freepik (https://www.freepik.com). The two-minute videos presenting the stimuli were created using Microsoft PowerPoint. Each video started with a black screen, while the title was being announced via audio alone (“How to repair an [air balloon or rocket ship]”). Next, a recorded statement said: “When [the balloon or rocket ship] is damaged, there are tools that can repair it”. The first stimulus then appeared on the screen, along with an audio stating, “This is a [object name]”. The next screen showed the object next to a broken part of the ship or balloon, with an audio indicating that the object is used to repair that part. Finally, an animation illustrated each object repairing the broken part with audio describing the actions. This sequence was identical for all objects.

### Behavioral gaze analysis

The recordings of the interactive sessions were manually annotated by an undergraduate student who was blind to the study topic and hypotheses. The analysis was conducted using MATLAB’s Video Labeler app. The coder indicated whether any of the following gaze behaviors occurred: (1) eye contact between the child and the instructor, (2) the child looking at the target stimulus, (3) the child and instructor looking at the same target stimulus, or (4) the child and instructor looking at the same irrelevant object. For each dyad and condition, we calculated the percentage of frames corresponding to each gaze behavior (continuous variable). Because mutual gaze toward the irrelevant object occurred extremely rarely (Scaffolding: 1.3 ± 1.5%, Passive: 0.2 ± 0.5%), we excluded this measure from further analyses. Paired *t*-tests were then used to compare the remaining measures between strategies. To determine whether gaze patterns predicted neural synchrony, as suggested by previous studies^50,51^, we computed Pearson correlations between each gaze measure and the child-instructor neural synchrony (Fisher’s *Z*-transformed) in each significantly coupled ROI. In addition, Pearson correlations were performed to examine the relationship between gaze patterns and learning outcomes. All statistical results were corrected for multiple comparisons using FDR^76^.

### Turn-taking analysis in the Scaffolding condition

Recordings of the Scaffolding sessions were manually transcribed and segmented. For each dyad, we calculated the number of speaking segments produced by each participant, their average duration, and the talk-time ratio between the instructor and the child. Child speaking segments were defined as segments containing vocal speech produced by the child. In addition, we quantified the number of questions the instructor asked the child, the number of answers provided, and vice versa, as well as the average number of questions per segment. Child answers included both attempted responses and explicit statements of uncertainty (e.g., “I don’t know”). Pearson correlations were performed to examine the relationship between turn-taking dynamics and learning outcomes. To determine whether turn-taking patterns predicted neural synchrony, as suggested by some studies^59,107^, we computed Pearson correlations between each measure and the child-instructor neural synchrony (Fisher’s *Z*-transformed) in each significantly coupled ROI. Results were FDR corrected^76^.

### fNIRS acquisition

A NIRSport2 device (NIRx) was used to record the neural activity of the dyads. A cap with 44 channels (16 sources and 15 detectors) was placed on the child and the instructor. The placement of the optodes was almost identical to the one used in a previous fNIRS brain-to-brain coupling study^54^, and was consistent with the international 10–20 system^108^ using MATLAB’s fOLD toolbox^109^ and NIR Site 2.0 software (NIRx). The channels were placed over the frontal, temporal, and parietal lobes. Within the frontal lobes, we focused on the prefrontal and premotor cortices, identifying four regions of interest (ROIs) in each hemisphere (eight ROIs in total; Fig. 1d. Full montage in Table S1). These eight ROIs covered cortical regions that are known to be associated with inter-subject correlation during social interaction and are considered to be part of the “social brain”, such as the default mode network regions (e.g., the ventromedial and dorsomedial prefrontal cortex, bilateral temporoparietal junction, and superior/inferior parietal lobe^34,35,66–68^), narrative comprehension regions (e.g., Broca’s areas, Wernicke’s areas^45,47^), and goal-directed attention regions (e.g., premotor, supplementary motor area- SMA^66,67,110^).

Eight short channels, four for each hemisphere, were used to remove superficial physiological noise. Near-infrared absorption rates (at two wavelengths: 760 and 850 nm) were measured at a sampling rate of 5.08 Hz. Oxyhemoglobin (HbO) and deoxyhemoglobin (HbR) concentrations were extracted using the modified Beer-Lambert law. The analysis was based on the HbO concentration, whose signal-to-noise ratio is better than HbR^111^, and has been used as an indicator to compute brain-to-brain coupling^55^. Nevertheless, we conducted child-instructor neural synchrony analysis on the HbR as well (Fig. S1).

### fNIRS preprocessing

Signals were pre-processed using Satori software (NIRx). A signal quality control was applied to each SNIRF file, where channels with a coefficient of variation exceeding 30% were excluded from the analysis. The raw wavelength data were converted into optical density. Then, short-channel regression (closest SSR) and spike removal were performed (Iteration:10; Lag: 5 s; Threshold: 3.50; Influencer: 0.50) and a Butterworth band pass filter of 0.5 to 0.01 Hz was applied. Finally, the optical density data were converted to concentration data and normalized with a z-transformation.

The STORM-Net co-registration tool was used to accurately estimate the channel position of the fNIRS cap placement on the subject’s head, based on a short video of the subject wearing the cap^112^. This application outputs the coordinates of each point of interest on the cap in a MNI coordinate system. For each subject, the channels were separated into ROIs based on their estimated coordinates. The data were averaged across channels within each ROI using Python 3.10.10. All subsequent analyses were based on these averaged signals and were conducted using Python and MATLAB.

### Child-child Inter-subject cross-correlation (ISC) analysis

The ISC analysis assessed the children’s brain activity while watching the videos. For each video, the neural response of each child was cross-correlated with the average neural response of the remaining (*N* − *1*) children for each ROI. The Pearson values were transformed into Fisher’s *Z*, yielding eight ISC scores per subject. To test ISC score significance, a null distribution of 1000 bootstrapped average ISC scores was generated for each ROI^84^. During each iteration, neural signals were transformed using a Fast Fourier Transform (FFT), thus preserving the amplitude spectrum while randomizing the phase independently for each subject but consistently across ROIs. The phase-scrambled signals were then inversed to the time domain, and the ISC was calculated by cross-correlating each subject’s original signal with the average phase-scrambled signal of the remaining group. The resulting ISC scores were averaged across subjects to produce a null distribution. Finally, the *p*-values for the empirical ISC scores were computed by comparing the average empirical scores to the null distribution. To examine the relationship between ISC and learning outcomes, Pearson correlations and linear least-squares regressions were performed for each strategy score and the ISC scores of each ROI for the corresponding video. We corrected for multiple comparisons using FDR^76^.

### Child-instructor cross-correlation analysis

The instructor’s signals from the interactive sessions were preprocessed as described above. For each interactive session of each dyad, a Pearson correlation was calculated between the child’s ROIs and the instructor’s ROIs, resulting in an 8×8 cross-correlation matrix. For each strategy, the 64 cross-correlation values were transformed using Fisher’s *R* to *Z* transformation. To compare overall child-instructor neural synchrony between conditions, *Z* values for all ROIs across dyads were obtained for each strategy. The cumulative distribution functions (CDFs) of these *Z* values were calculated for both the Scaffolding and the Passive sessions, representing the likelihood of obtaining each correlation value or lower within each strategy. We used a Kolmogorov-Smirnov test to examine whether these distributions were equal.

To identify paired ROIs that were significantly correlated, we compared the subjects’ *Z* values in each paired ROI to 0 using a one sample *t*-test^113,114^. Multiple comparisons were corrected using a bootstrap hypothesis test^115^. In each iteration, we randomly paired children and instructors, calculated the Pearson correlations for ROIs that were significantly coupled in the original pairs, transformed these correlations into Fisher’s *Z* values, and tested them against 0 using a one sample *t*-test. This process was repeated 1000 times to create a null distribution for each coupled ROIs. Then, we verified whether the empirical *t*-statistics were within the top 5% of the largest values in their respective null distributions. Finally, to evaluate whether child-instructor neural synchrony predicted children’s outcomes, we conducted a Pearson correlation analysis between the children’s scores and the child-instructor cross-correlation values across all significantly coupled ROIs.

To determine whether each learning strategy had a distinct synchrony pattern, we conducted a d-prime-based classification test. We first calculated the Euclidean distance between the cross-correlation matrix of each dyad and the group-averaged matrix while excluding that dyad. Thus, the group-averaged matrix was derived either from the same condition as the dyad or from the alternative condition. These distances were used to create a d-prime classifier. The “hit” rate was calculated as the proportion of instances where the distance between a Scaffolding pair and the Scaffolding group was smaller than the distance between the same pair and the Passive group. The “false alarm” rate was calculated as the proportion of instances where the distance between a Passive pair and the Scaffolding group was smaller than the distance between the same pair and the Passive group.

To examine potential lagged cross-correlations, the child’s time course was shifted relative to the instructor’s from −20 to 20 s in 1-second increments for each dyad and each strategy. At each shifted time point, Pearson correlations were calculated for significantly coupled ROIs and transformed into Fisher’s *Z* values. These values were averaged across dyads, and the time point of peak synchrony was identified for each coupled ROI. Compared to the non-shifted time point, a positive shift indicated that the child was leading, whereas a negative shift indicated that the instructor was leading.

### Remote experiment

A remote experiment was conducted to assess the importance of the face-to-face experience on the effects of learning strategies. Twenty-nine children were recruited, following the same assent and consent process as described above. Five children were excluded from the final analysis: three due to technical difficulties and two because of significant time gaps between conditions. The remaining 24 participants (age 6.69 ± 0.34 years, 11 females) were included in the analysis. The procedure and materials were identical to those of the main experiment, except for the experimental settings. The experiment was conducted remotely, with children viewing the videos and interacting with the instructors via Zoom software. The order of conditions was counterbalanced across children. Session length did not differ by presentation order [1^st^ session: 139.7 ± 13.2 s vs. 2^nd^ session: 139.3 ± 9.6 s, *t*(23) = 0.12, *p* = 0.9]. Two instructors conducted the sessions; session length and learning outcomes did not differ between instructors. Only behavioral measurements were collected during the remote experiment (the test following the interactive learning phase), without any cortical recordings or gaze-tracking data.

## Supplementary information

Supplementary Information

## Data Availability

All the data and the materials (except for the participants’ videos) have been deposited on the Open Science Framework and are publicly available at: 10.17605/OSF.IO/DBW6C.

## Code availability

Code for all analyses (Python and MATLAB) is publicly available on the Open Science Framework at: 10.17605/OSF.IO/DBW6C.
