# Supplementary material for: Enhanced word learning and neural synchrony during children’s storytelling
Source: NPJ Sci Learn. 2026 Mar 28;11:35. doi: 10.1038/s41539-026-00417-7 (PMC13246770; doi:10.1038/s41539-026-00417-7)
Supplement: Supplementary file 1 — Supplementary Information [file 41539_2026_417_MOESM1_ESM.pdf]

Supplementary Information

Table S1. Optode locations based on the 10-20 system, their assigned regions of interest, and the corresponding Brodmann areas for each channel used in this study (including short channels), generated by using MATLAB's fOLD toolbox[1].

Channels	*Optodes positions (10-20 system)* Sources	Detectors	*Region of interest (ROI)*	*Brodmann area (BA)*
1	F3	F5	Left prefrontal cortex	Dorsolateral prefrontal cortex (46)
2	F3	FC3	Left prefrontal cortex	Dorsolateral prefrontal cortex (9)
3	FC5	F5	Left prefrontal cortex	Broca's area *(44)*
4	FC5	FC3	Left prefrontal cortex	Broca's area *(44)*
5	FC5	C5	Left premotor cortex	Subcentral area *(43)*
7	FC1	FC3	Left prefrontal cortex	Dorsolateral prefrontal cortex (9)
8	FC1	C1	Left premotor cortex	Premotor and Supplementary Motor cortex (SMA) *(6)*
10	T7	C5	Left temporal lobe	Superior temporal gyrus *(22)*
11	T7	TP7	Left temporal lobe	Inferior temporal gyrus *(20)*
12	C3	FC3	Left premotor cortex	Premotor and SMA *(6)*
13	C3	C5	Left premotor cortex	Premotor and SMA *(6)*
14	C3	C1	Left premotor cortex	Premotor and SMA *(6)*
15	C3	CP3	Left parietal lobe	Supramarginal gyrus (Wernicke's area) *40*
16	CP5	C5	Left temporal lobe	Superior temporal gyrus *(22)*
17	CP5	TP7	Left temporal lobe	Superior temporal gyrus *(22)*
18	CP5	CP3	Left parietal lobe	Angular gyrus (Wernicke's area) *(39)*
19	CP5	P5	Left temporal lobe	Angular gyrus (*39)*
21	CP1	P1	Left parietal lobe	Somatosensory Association cortex (7)
22	CP1	C1	Left parietal lobe	Primary Somatosensory cortex (3)
23	CP1	CP3	Left parietal lobe	Supramarginal gyrus (40)
25	P3	P1	Left parietal lobe	Somatosensory Association cortex (7)
26	P3	CP3	Left parietal lobe	Angular gyrus (39)
27	P3	P5	Left parietal lobe	Angular gyrus (*39)*
28	F4	F6	Right prefrontal cortex	Dorsolateral prefrontal cortex (46)
29	F4	FC4	Right prefrontal cortex	Dorsolateral prefrontal cortex (9)
30	FC6	F6	Right prefrontal cortex	Inferior frontal gyrus *(45)*
31	FC6	FC4	Right prefrontal cortex	Inferior frontal gyrus *(44)*
32	FC6	C6	Right premotor cortex	Subcentral area *(43)*
34	FC2	FC4	Right prefrontal cortex	Dorsolateral prefrontal cortex (9)
35	FC2	C2	Right premotor cortex	Premotor and SMA *(6)*
37	T8	C6	Right temporal lobe	Superior temporal gyrus *(22)*
38	T8	TP8	Right temporal lobe	Inferior temporal gyrus *(20)*
39	C4	FC4	Right premotor cortex	Premotor and SMA *(6)*
40	C4	C6	Right premotor cortex	Premotor and SMA *(6)*
41	C4	C2	Right premotor cortex	Premotor and SMA *(6)*
42	C4	CP4	Right parietal lobe	Supramarginal gyrus (*40)*
43	CP6	C6	Right temporal lobe	Superior temporal gyrus *(22)*
44	CP6	TP8	Right temporal lobe	Superior temporal gyrus *(22)*
45	CP6	CP4	Right parietal lobe	Angular gyrus (*39)*
46	CP6	P6	Right temporal lobe	Angular gyrus (*39)*
48	CP2	C2	Right parietal lobe	Primary Somatosensory cortex (3)

49	CP2	CP4	Right parietal lobe	Supramarginal gyrus (40)
51	P4	CP4	Right parietal lobe	Angular gyrus (*39)*
52	P4	P6	Right parietal lobe	Angular gyrus (*39)*
6	FC5	---	Short channel	---
9	FC1	---	Short channel	---
20	CP5	---	Short channel	---
24	CP1	---	Short channel	---
33	FC6	---	Short channel	---
36	FC2	---	Short channel	---
47	CP6	---	Short channel	---
50	CP2	---	Short channel	---

a. Scaffolding

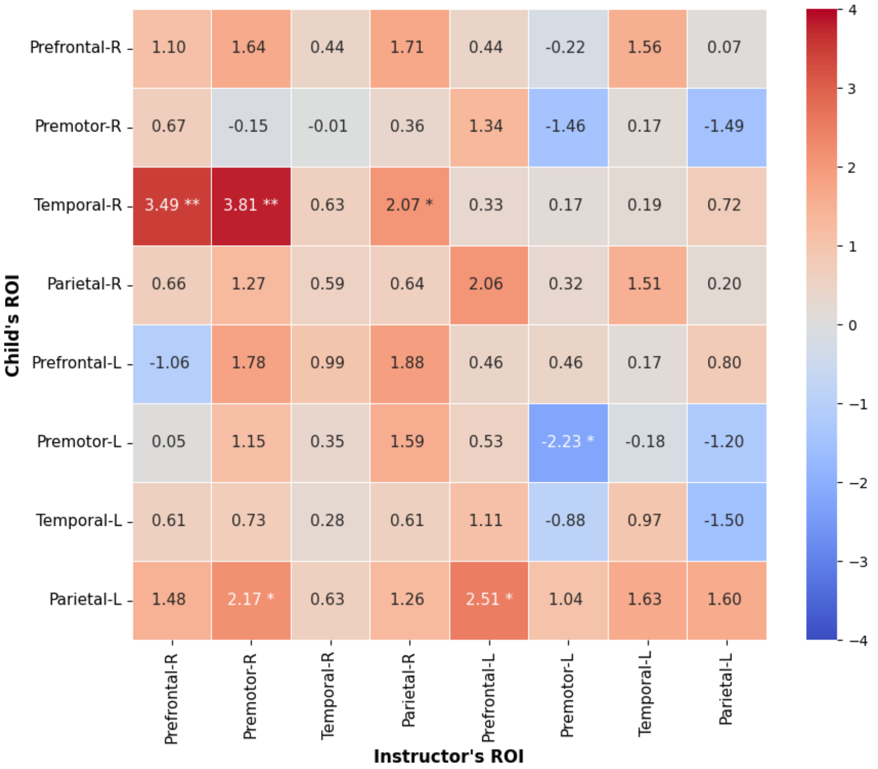

b. Passive

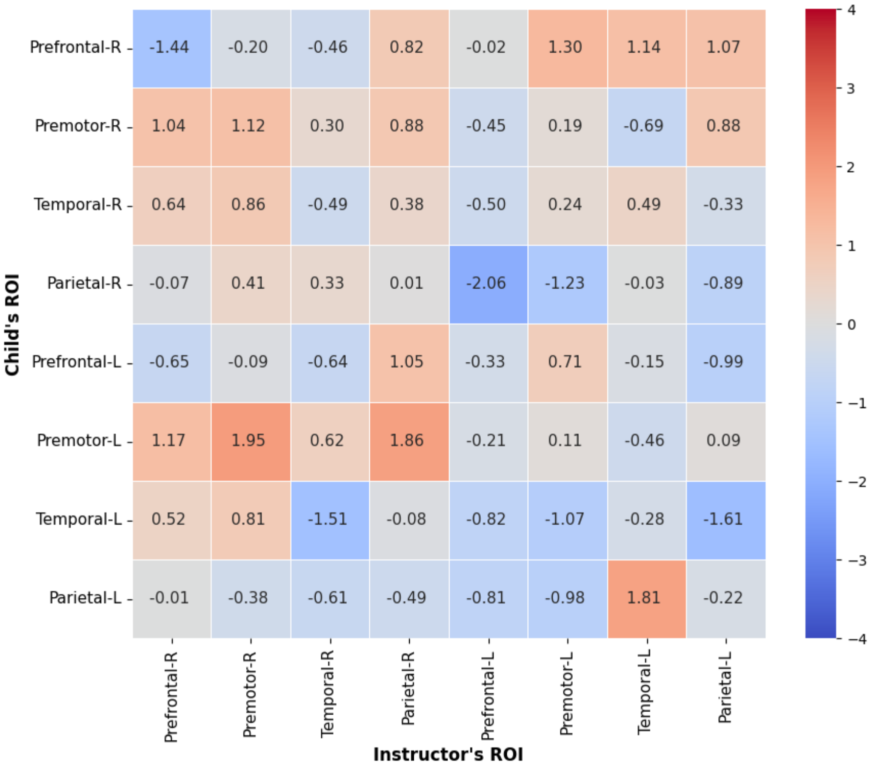

Figure S1. Analysis of child-instructor neural synchrony based on HbR concentration during the Scaffolding (a) and the Passive (b) sessions. Rows and columns represent children's and instructor's ROIs, respectively (R - right, L - left). The values shown are t-statistics from one-sample t-tests comparing the Fisher Z values of the Pearson correlations to zero. In Scaffolding, there were six significantly coupled regions, whereas no significant coupling was observed in Passive. (* = $p < 0.05$, ** = $p < 0.005$)

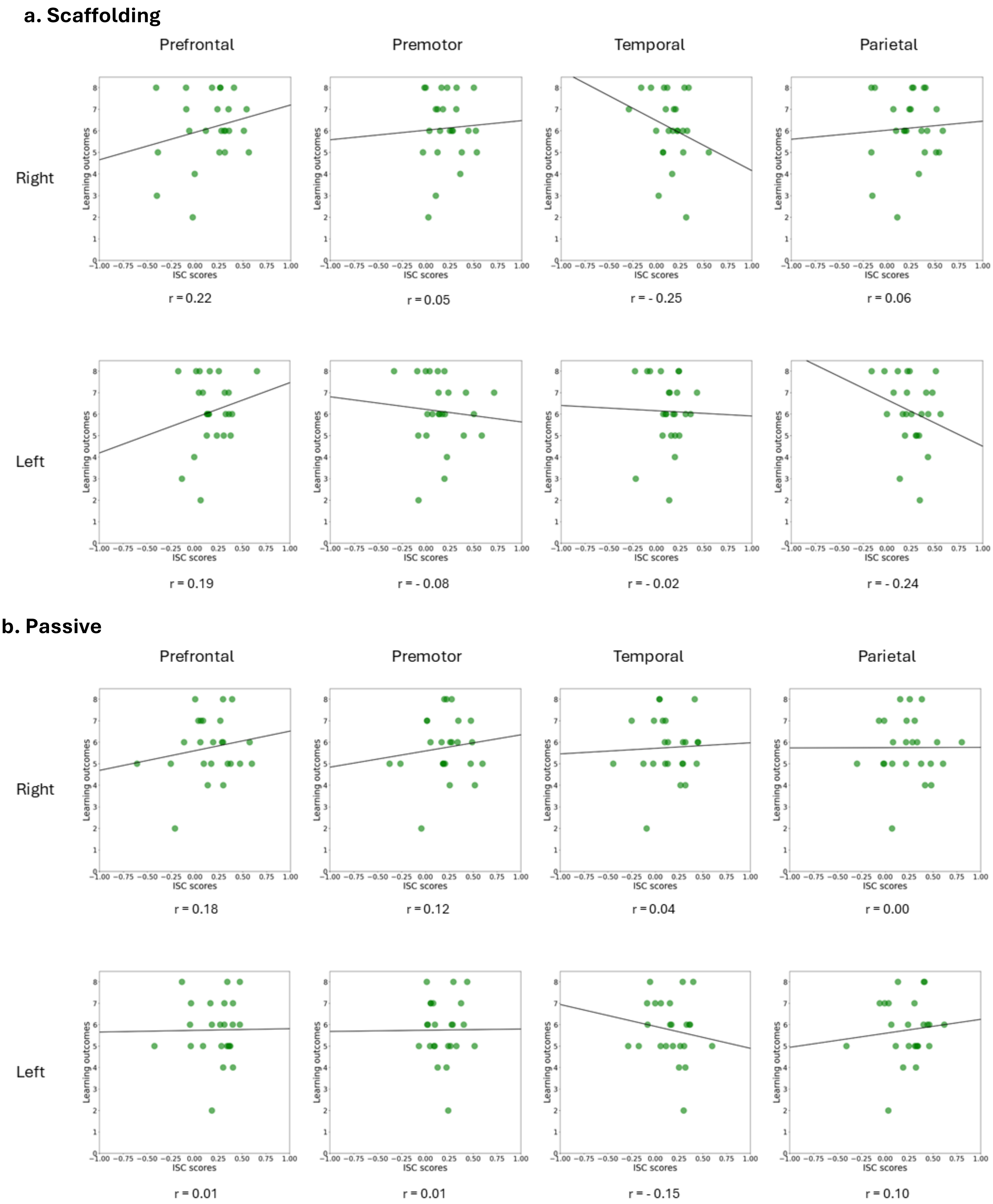

Figure S2: **Correlations between learning outcomes and child-child ISC scores during video watching in Scaffolding (a) and Passive (b).** No correlation was found in any ROI for either strategy.

a. Scaffolding

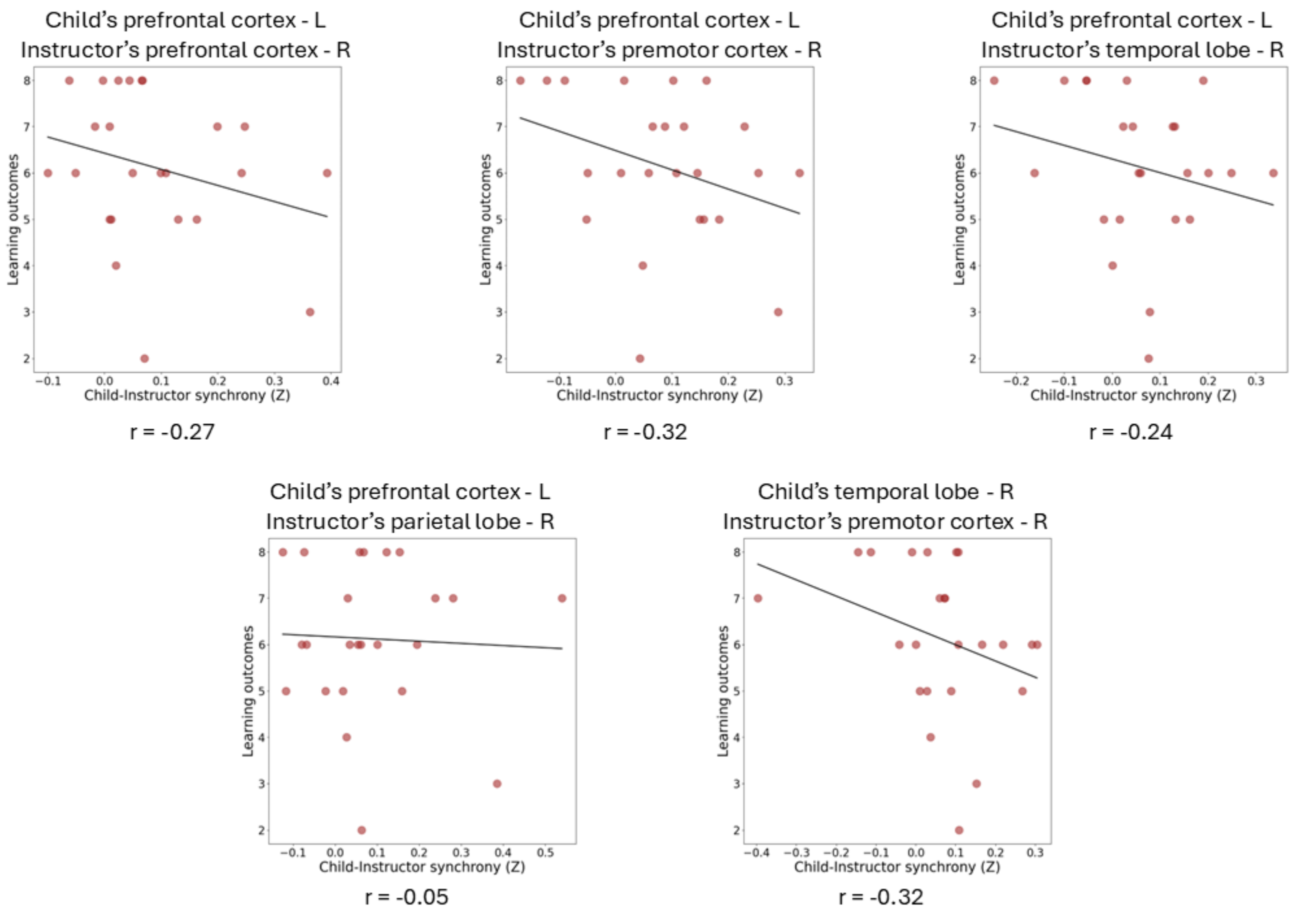

b. Passive

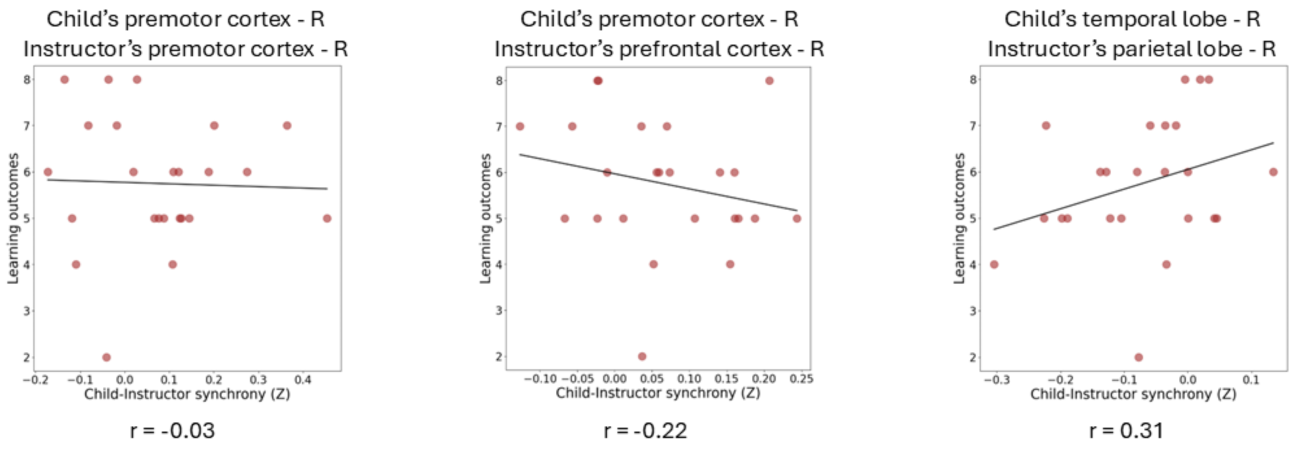

Figure S3. **Correlations between children’s scores and child-instructor neural synchrony, in the Scaffolding (a) and the Passive (b) sessions**. Within the significantly coupled ROIs, child-instructor neural synchrony did not predict children's performance in either session.

a. Scaffolding

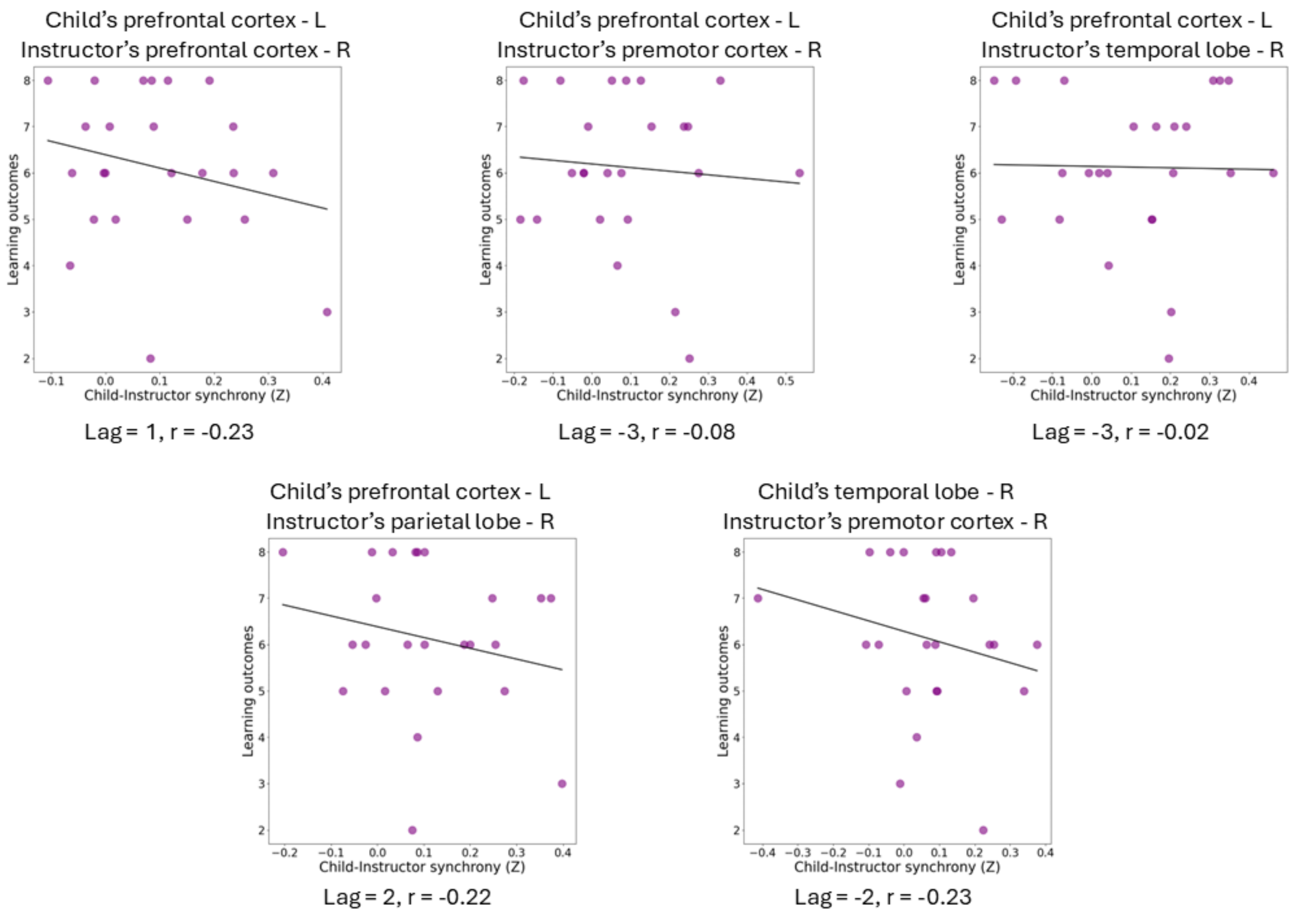

b. Passive

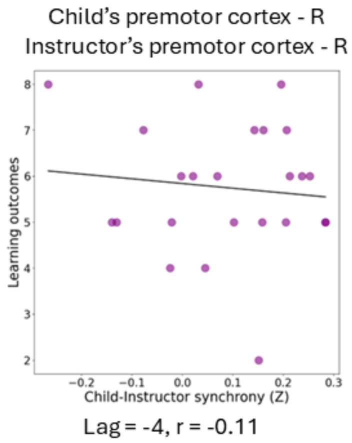

Figure S4. **Correlations between children's scores and child-instructor lagged synchrony in the Scaffolding (a) and the Passive (b) sessions**. For each significantly coupled ROI (R - right, L - left) where a lagged correlation was detected, we tested whether adjusting the synchrony for this lag would better predict learning outcomes. However, neural synchrony remained non-predictive of learning outcomes for both sessions.

References

1. Zimeo Morais, G. A., Balardin, J. B. & Sato, J. R. fNIRS Optodes' Location Decider (fOLD): a toolbox for probe arrangement guided by brain regions-of-interest. *Sci. Rep.* **8**, 3341 (2018).
